# The complete mitochondrial genome of *Sinocyclocheilus aluensis* (Cyprinoidea: Cyprinidae)

**DOI:** 10.1080/23802359.2020.1756482

**Published:** 2021-02-15

**Authors:** Lili Cui, Xiangjun Miao, Yanchun Yang, Cuilian Zheng, Chunyun Lei, Mingli Li, Guanghua Li, Haitao Gao, Xiaoyi Wang, Shaoxiong Lu

**Affiliations:** a College of Animal Science and Technology, Yunnan Agricultural University, Kunming, PR China; b Yunnan Academy of Fishery Sciences, Kunming, PR China; c Garden Management Station, Housing and Urban-rural Development Bureau of Luxi County, Luxi, PR China; d Government Investment Audit Center, Luxi County Audit Bureau, Luxi, PR China

**Keywords:** *Sinocyclocheilus aluensis*, mitochondrial genome, phylogeny

## Abstract

We describe the complete mitochondrial genome of the endangered fish *Sinocyclocheilus aluensis*. It is a circular molecule of 16,583 bp in size with a D-loop region and contains 22 transfer RNA (tRNA) genes, 2 ribosomal RNA (rRNA) genes and 13 protein-coding genes, and all genes show the typical gene arrangement conforming to the vertebrate consensus. The overall base composition of *S*. *aluensis* mitogenome is 31.1% for A, 26.9% for C, 16.6% for G, and 25.4% for T. The percentage of G + C content is 43.5%. Phylogenetic analysis demonstrated that all *Sinocyclocheilus* species clustered together and formed a monophyletic group. The mitochondrial genome sequencing for *S*. *aluensis* in this study provides important molecular data for further evolutionary research for *Sinocyclocheilus*.

*Sinocyclocheilus aluensis*, which belongs to order Cyprinoidea, family Cyprinidae, genus *Sinocyclocheilus*, is an extremely precious species of freshwater fish, which is only found in Luxi area, Yunnan Province, China. It was previously synonymized with *S. angustiporus* (Zheng and Xie 1985), but was corrected to a valid species in 2005 (Li et al. 2005).

In this study, we determined the complete mtDNA sequence of *S. aluensis*. Samples were collected from Luxi county of Yunnan Province in China (24°39′N; 103°58′E). The specimen is stored in the Specimen Museum of Yunnan Academy of Fishery Sciences and its accession number is 20180819001. The sequencing results were assembled using GetOrganelle. Genomes were predicted using the MitoAnnotator (http://mitofish.aori.u-tokyo.ac.jp/annotation/input.html). The transfer RNA (tRNA) genes were also identified using the MitoAnnotator. The locations of protein-coding genes were determined by comparing with the corresponding known sequences of other *Sinocyclocheilus* fish species.

The whole mitochondrial genome length of *S. aluensis* was 16,583 bp in length (GenBank accession number MT122746). It consisted of a non-coding control region (D-loop), 13 protein-coding genes, 2 ribosomal RNA genes (rRNAs), and 22 transfer RNAs (tRNAs). The contents of A, C, G, and T were 31.1%, 26.9%, 16.6% and 25.4%. The percentage of G + C content was 43.5%, which was lower than that of A + T content (56.5%). To demonstrate the phylogenetic position of *S. aluensis*, we performed MEGA 7.0 (Kumar et al. 2016) to align all selected sequences and constructed a neighbour-joining tree containing complete mitochondrial genome DNA of 18 species. The results from the phylogenetic analysis revealed that all *Sinocyclocheilus* species clustered together and formed a monophyletic group, and *S. aluensis* has a close relationship with *S. oxycephalus* (Figure 1).

**Figure 1.**
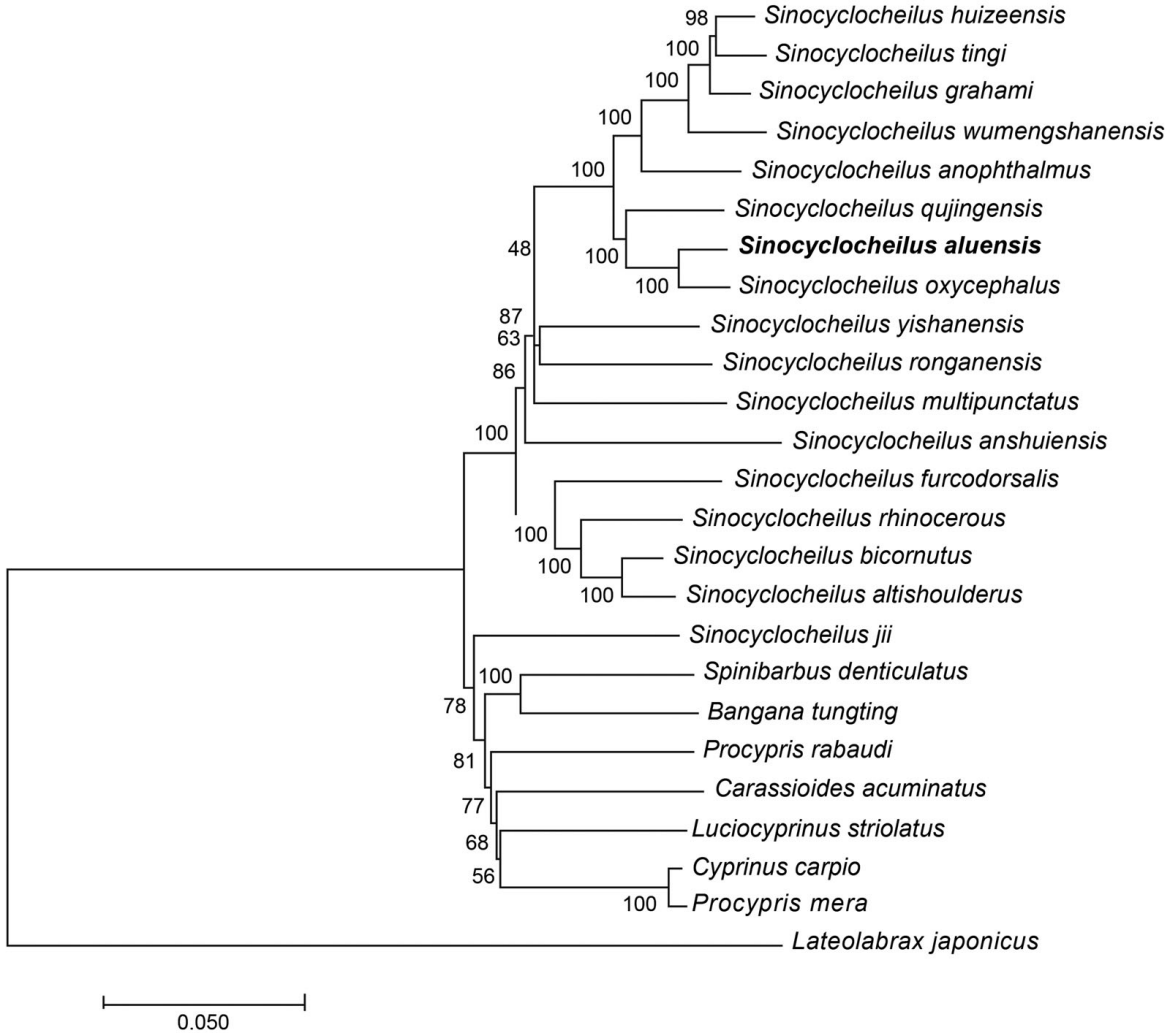
A neighbour-joining (NJ) tree of the 19 species from Cyprinoidea was constructed based on complete mitochondrial genome data. The analyzed species and corresponding NCBI accession numbers are as follows: *Sinocyclocheilus huizeensis* (NC_044072.1), *Sinocyclocheilus tingi* (NC_039594.1), *Sinocyclocheilus graham* (GQ148557.1), *Sinocyclocheilus wumengshanensis* (NC_039769.1), *Sinocyclocheilus anophthalmus* (KF892542.1), *Sinocyclocheilus qujingensis* (NC_043910.1), *Sinocyclocheilus aluensis* (MT122746), *Sinocyclocheilus oxycephalus* (NC_037858.1), *Sinocyclocheilus yishanensis* (MK387704.1), *Sinocyclocheilus ronganensis* (KX778473.1), *Sinocyclocheilus multipunctatus* (MG026730.1), *Sinocyclocheilus furcodorsalis* (GU589570.1), *Sinocyclocheilus rhinocerous* (KR069119.1), *Sinocyclocheilus bicornutus* (KX528071.1), *Sinocyclocheilus altishoulderus* (FJ984568.1), *Sinocyclocheilus anshuiensis* (KR069120.1), *Cyprinus carpio* (KU159761.1), and *Lateolabrax japonicas* (KR780682.1).

## Data Availability

The data that support the findings of this study are openly available in Nucleotide at https://www.ncbi.nlm.nih.gov/, reference number [NC_044072.1, NC_039594.1, GQ148557.1, NC_039769.1, KF892542.1, NC_043910.1, MT122746, NC_037858.1, MK387704.1, KX778473.1, MG026730.1, GU589570.1, KR069119.1, KX528071.1, FJ984568.1, KR069120.1, KU159761.1, and KR780682.1].
